# CircAkap7: A novel circular RNA with a role in Doxorubicin-induced cardiotoxicity

**DOI:** 10.1016/j.gendis.2024.101253

**Published:** 2024-03-02

**Authors:** Jiaqin Liu, Sa Liu, Xiangyun Li, Shanshan Wei, Taoli Sun, Ning Xie, Bikui Zhang, Wenqun Li

**Affiliations:** a Department of Pharmacy, The Second Xiangya Hospital, Central South University, Changsha, Hunan 410011, China; b Institute of Clinical Pharmacy, Central South University, Changsha, Hunan 410011, China; c Department of Pharmacy, Jianli People's Hospital, Jingzhou, Hubei 433300, China; d School of Pharmacy, Hunan University of Chinese Medicine, Changsha, Hunan 410208, China; e Department of Breast Cancer Medical Oncology, Hunan Cancer Hospital, Changsha, Hunan 410013, China

Doxorubicin (Dox), an anthracycline chemotherapy drug, is used extensively in cancer chemotherapy and has been studied in depth in relation to cancers including leukemia, small-cell lung cancer, and ovarian cancer.^1^ However, the dose-dependent cardiotoxicity of Dox seriously limits its clinical application.^2^ As a type of noncoding RNA with a covalent cyclic structure, circular RNA (circRNA) has emerged as a new and exciting topic. Growing evidence has shown that circRNA is linked to the pathogenesis of various cardiovascular diseases, including Doxorubicin-induced cardiotoxicity (DIC).^3^ The function of circRNA is closely associated with its cellular sublocalization. CircRNAs distributed in the cytoplasm primarily serve as microRNA (miRNA) sponges that competitively adsorb miRNA and reduce the binding and degradation of the target mRNA by miRNA, leading to indirect up-regulation of the target gene and increased execution of its function.^4^ In the present research, a novel circRNA named mmu_circ_0000153 with a length of 840 bp was identified in DIC through circRNA sequencing. It is derived from the precursor Akap7 mRNA through the back-splicing of exons and is therefore also referred to as circAkap7. Functionally, circAkap7 serves as an endogenous protective factor for the heart, as it was activated and increased in abundance to resist Dox-stimulated cardiomyocyte damage, whereas its knockdown intensified Dox-induced cardiomyocyte apoptosis. To examine the mechanism, the circAkap7‒miRNA‒mRNA (messenger RNA) ceRNA (competing endogenous RNA) network was constructed through miRNA prediction and mRNA sequencing based on circAkap7 knockdown.

Echocardiography (Fig. 1A), slice staining, and the detection of myocardial injury markers (Fig. S1A–C) confirmed the successful construction of the DIC mouse model. Then, circRNA sequencing was performed on the control group and the Dox group of the DIC model, and a total of 11,418 circRNAs were detected. According to the screening criteria “|log_2_ Fold change (FC)| > 1, *p* < 0.05”, 41 differentially expressed circRNAs were identified, of which 17 were up-regulated and 24 were down-regulated (Fig. 1B; S1D). Quantitative reverse transcription PCR verified that CircAkap7 was significantly increased in the DIC cell and mouse models, which was consistent with the sequencing results (Fig. 1C, D; S2 and Table S1).

**Figure 1:**
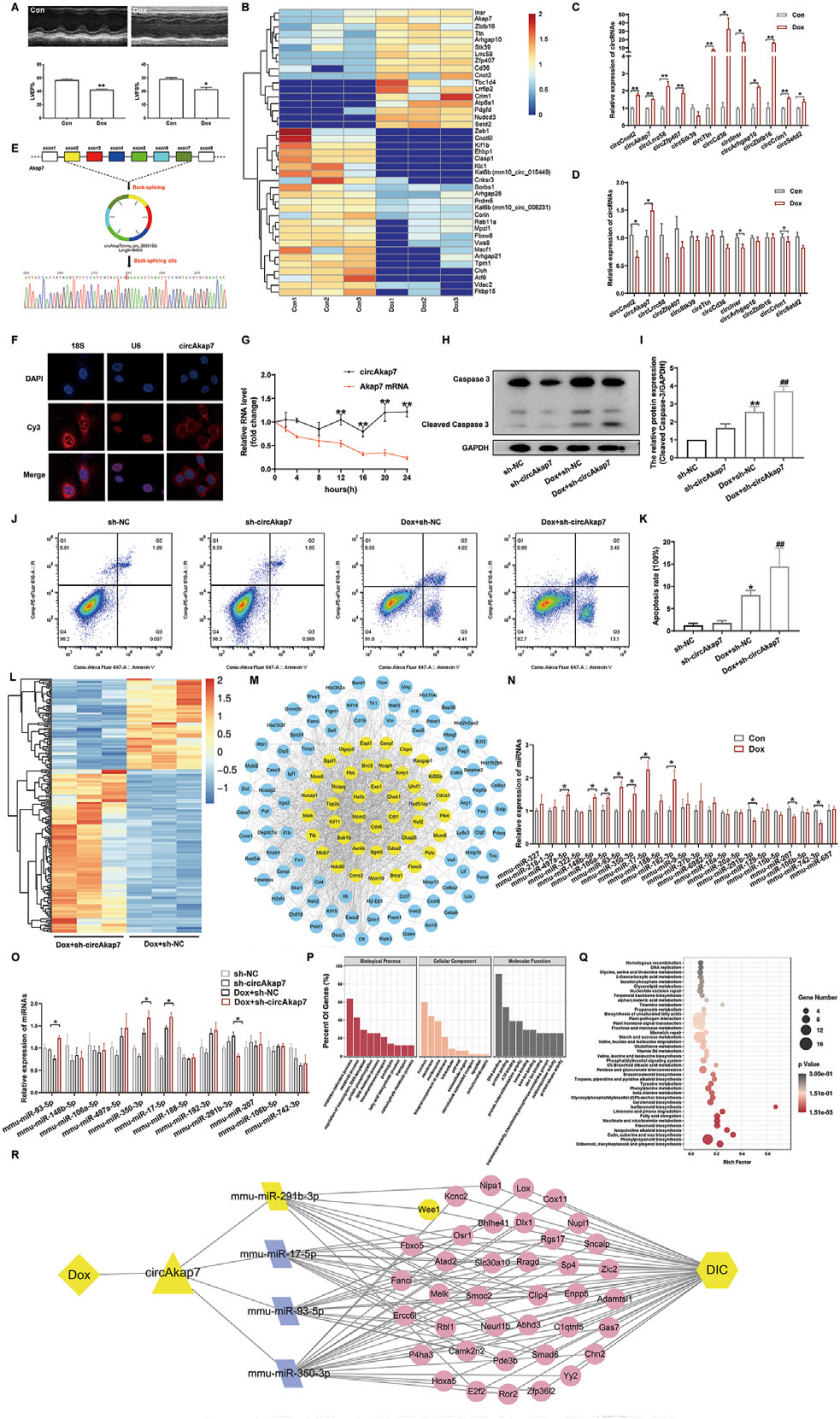
The protective role of novel circAkap7 in Dox-induced cardiomyocyte apoptosis and the construction of circAkap7‒miRNA‒mRNA ceRNA network. **(A)** Representative captures of echocardiography and the statistical charts of left ventricular ejection fraction (LVEF%) and left ventricular ejection fraction (LVFS%). ∗*p* < 0.05, ∗∗*p* < 0.01 *vs*. Con. **(B)** Cluster heat map of differentially expressed circRNAs. The rows represent circRNAs and the columns represent samples. A change in color from blue to red notes the expression level of the gene from low to high. **(C)** RT-qPCR verified the differential expression of up-regulated circRNAs in the DIC cell model (*n* = 3). ∗*p* < 0.05, ∗∗*p* < 0.01 *vs*. Con. **(D)** RT-qPCR verified the differential expression of up-regulated circRNAs in the DIC mouse model (*n* = 10). ∗*p* < 0.05, ∗∗*p* < 0.01 *vs*. Con. **(E)** Sanger sequencing validated the black-splicing sequence of circAkap7. **(F)** FISH assay. Nuclei appear in blue fluorescence and circRNAs appear in red fluorescence. **(G)** RT-qPCR examined the levels of circRNAs and mRNA in actinomycin D-treated HL-1 cells. circAkap7 is more stable than Akap7 mRNA. **(H)** Relative expression of cleaved caspase-3 protein. **(I)** Expression level of cleaved caspase-3. GAPDH was the loading control. ∗*p* < 0.05, ∗∗*p* < 0.01 *vs*. sh-NC; #*p* < 0.05, ##*p* < 0.01 *vs*. Dox + sh-NC; *n* = 3. **(J)** Annexin V (propidium iodide and fluorescein isothiocyanate-conjugated) staining detected cardiomyocyte apoptosis rate in cells with circAkap7 knockdown. **(K)** The statistic graph of the Annexin V staining. ∗*p* < 0.05, ∗∗*p* < 0.01 *vs*. sh-NC; #*p* < 0.05, ##*p* < 0.01 *vs*. Dox + sh-NC; *n* = 3. **(L)** Cluster heat map of differentially expressed mRNAs. The rows represent mRNAs and the columns represent samples. A change in color from blue to red notes the expression level of the gene from low to high. **(M)** The protein–protein interaction network with 127 nodes and 1344 edges extracted from Fig. S6B using the CytoNCA plugin. **(N)** The expression of potential target microRNAs in the Con group and Dox group detected by RT-qPCR. **(O)** The expression of potential target microRNAs in the Con group and Dox group detected by RT-qPCR. **(P)** GO functional enrichment analysis. **(Q)** KEGG pathway analysis. **(R)** circAkap7‒miRNA‒mRNA regulatory network in DIC. The diamond represents Dox, the triangle represents circAkap7, the parallelograms represent miRNAs, the ellipses represent mRNAs, and the hexagon represents DIC. Nodes in yellow represent the Dox/circAkap7/mmu-miR-291b-3p/Wee1/Dox-induced cardiotoxicity axis. Dox, Doxorubicin; RT-qPCR, quantitative reverse transcription PCR; DIC, Doxorubicin-induced cardiotoxicity.

Considering that circAkap7 (mmu_circ_0000153) was discovered through circRNA sequencing and has not been previously reported, we performed cyclic identification to further validate its circular structure. Sanger sequencing (Fig. 1E) revealed that circAkap7 had a back-splicing sequence, which was further confirmed by agarose gel electrophoresis experiments (Fig. S3B and Table S3) demonstrating its cyclic structure. Actinomycin D is widely used in RNA stability determination to inhibit RNA transcription. Actinomycin D experiments clarified that circAkap7 had a longer half-life than its corresponding mRNA (Fig. 1G). Additionally, experiments involving RNase R digestion (Fig. S3D) revealed that circAkap7 was more resistant to RNase R enzyme digestion, indicating a stable circular structure of circAkap7.

To explore the function of circAkap7 in DIC, circAkap7 knockdown cell lines were constructed using lentiviral vectors (Fig. S4A), and the knockdown efficiency was verified by quantitative reverse transcription PCR (Fig. S4B, C and Table S4). The results from a CCK-8 experiment (Fig. S4D) demonstrated that knocking down circAkap7 intensified Dox-induced cardiomyocyte apoptosis, contrary to our initial expectation, which led us to speculate that circAkap7 may function as an endogenous protective factor. There are endogenous protective factors present in the myocardium, which are activated or up-regulated to counteract cardiac injury when the heart is damaged. However, if the activated or up-regulated endogenous protective factors are insufficient to counter the damage, additional cardiac injury occurs.^5^ Moreover, in our study, Western blot experiments (Fig. 1H, I) and flow cytometry (Fig. 1J, K) proved the worsening effect of circAkap7 knockdown in DIC. Therefore, we concluded that circAkap7 serves as an endogenous cardioprotective factor whose abundance was increased to counteract the cardiac damage caused by Dox, whereas knockdown of circAkap7 amplified Dox-induced cell apoptosis.

We performed mRNA sequencing on Dox-treated sh-NC cells and Dox-treated sh-circAkap7 cells to further investigate the mechanism of circAkap7 in regulating DIC. The principal component analysis shown in Figure S5A and the heatmap shown in Figure S5C suggested good repeatability of samples within the group and a significant difference. With the defined threshold value (|log_2_ FC| > 0.585, *p* < 0.05), a total of 1510 differentially expressed mRNAs were screened, of which 979 were significantly up-regulated and 531 were down-regulated (Fig. 1L; Fig. S5B). Next, we performed GO, KEGG, and GSEA analyses of the differentially expressed mRNAs, which showed that the differentially expressed mRNAs were mainly enriched in RNA polymerase II's positive regulation for transcription, DNA replication, the Wnt signaling pathway, the TNF signaling pathway, the IL-17 signaling pathway, and hypertrophic cardiomyopathy (Fig. S5D–F), indicating that circAkap7 knockdown could augment DIC through diverse genes and multiple pathways.

In addition, we constructed a protein–protein interaction network utilizing the data of 1510 differentially expressed genes. Three Cytoscape plugins (CytoNCA, CytoHubba, and MCODE) were selected to perform topology analysis of the protein–protein interaction network and to mine the core network and core protein. Finally, we obtained 3 core protein–protein interactions (Fig. S6C‒E), 1 secondary core network (Fig. 1M), and 32 core genes (Fig. S6H) that are potentially involved in the exacerbation of DIC through the knockdown of circAkap7. A larger node degree value corresponds to a more important role of the node. Furthermore, we calculated the degree distribution of the protein–protein interaction network in Figure S6 and the node degree value of the core protein–protein interactions (Fig. S7A–J).

Different cell sublocations of circRNAs usually contribute to different biological functions. Most cytoplasmic circRNAs can regulate downstream mRNAs through binding to miRNAs via the ceRNA mechanism. FISH localization experiments (Fig. 1F) and nucleoplasmic separation (Fig. S3C) determined that circAkap7 was mainly distributed in the cytoplasm, regulating downstream target genes through a sponge mechanism. Therefore, we predicted the potential target miRNAs of circAkap7 through two online databases, miRanda and miRDB, and obtained 253 miRNAs. After literature screening, 23 miRNAs related to cardiovascular disease were selected for experimental verification (Fig. S8A). Quantitative reverse transcription PCR showed that four miRNAs were differentially expressed in Dox-treated cardiomyocytes and Dox-treated circAkap7 knockdown cardiomyocytes: mmu-miR-93-5p, mmu-miR-350-3p, mmu-miR-17-5p, and mmu-miR-291b-3p (Fig. 1N, O and Table S2). The binding of circAkap7 to mmu-miR-93-5p, mmu-miR-350-3p, mmu-miR-17-5p, and mmu-miR-291b-3p was observed as depicted in Figure S8B.

The potential target mRNAs of the four miRNAs were predicted through TargetScan and miRDB online databases. The intersection of the results revealed 706, 1240, 705, and 693 target mRNAs for mmu-miR-93-5p, mmu-miR-350-3p, mmu-miR-17-5p, and mmu-miR-291b-3p, respectively (Fig. S8C–F). Consistent with the trends shown by its target mRNAs, a circRNA can positively regulate its target mRNA by acting as a miRNA sponge. To further screen the target mRNAs regulated by circAkap7 and the 4 miRNAs at the same time, we took the intersection of the target mRNAs of the predicted miRNAs with the sequenced mRNAs that were significantly down-regulated after circAkap7 knockdown (Fig. S8G–J). Therefore, we constructed a circAkap7‒miRNA‒mRNA ceRNA network (Fig. 1R), including 1 circRNA (circAkap7), 4 miRNAs (mmu-miR-93-5p, mmu-miR-350-3p, mmu-miR-17-5p, and mmu-miR-291b-3p), and 39 mRNAs. GO function enrichment analysis (Fig. 1P) and KEGG pathway analysis (Fig. 1Q) were performed on the 39 mRNAs in the network constructed above, and the results were consistent with the pathways enriched in the sequenced mRNAs. These results demonstrated that circAkap7 is involved in the development of DIC through the miRNA‒mRNA axis. Among 39 mRNAs, only the Wee1 gene was identified as a core gene in the protein–protein interaction screening (Fig. 1M). In addition, mmu-miR-291b-3p was the only miRNA in the ceRNA network that interacted with Wee1. Thus, we hypothesized that the circAkap7/mmu-miR-291b-3p/Wee1 axis (highlighted in yellow in Fig. 1R) may play a crucial role in the pathogenesis and progression of DIC.

Currently, we have made preliminary progress in unveiling the function and mechanism of circAkap7 in DIC through *in vitro* experiments. We will continue to validate its functional mechanism *in vivo* in the future.

In conclusion, we elucidated the ceRNA mechanism by which circAkap7 aggravates DIC, providing a novel research target and solution for the treatment of DIC. CircAkap7 is an endogenous cardioprotective factor whose knockdown exacerbated the cardiotoxicity of Dox, mainly through the regulation of the miRNA/mRNA axis. The combination of circRNA sequencing, mRNA sequencing, and target prediction from databases contributed to constructing the circAkap7‒miRNA‒mRNA ceRNA network, within which circAkap7/mmu-miR-291b-3p/Wee1 was most likely to participate in regulating DIC.

## Ethics declaration

The Medicine Animal Welfare Committee of Xiangya School of Medicine approved the experimental protocol with the ethics approval number CSU-2022-0260. All animal experiments were executed in accordance with the National Institutes of Health Guide (NIH Publications No. 8023) for the Care and Use of Laboratory Animals.

## Author contributions

WL and JL designed the research. JL, SL, XL, SW, and TS completed the entire research. SL wrote the manuscript. TS, JL, and WL participated in the manuscript editing. NX, BZ, and WL provided the experimental funding. All the authors read and approved the final manuscript.

## Conflict of interests

The authors declare that they have no known competing financial interests or personal relationships that could have appeared to influence the work reported in this paper.

## Funding

This study was supported by grants from the 10.13039/501100001809National Natural Science Foundation of China (No. 82173911, 81973406, 82373970), Hunan Provincial Natural Science Foundation of China (No. 2023JJ30761, 2023JJ30802), Scientific Research Project of Hunan Provincial Health and Family Planning Commission (China) (No. 202113050843), Research Project established by Chinese Pharmaceutical Association Hospital Pharmacy Department (No. CPA-Z05-ZC-2021-002), 10.13039/501100019054Changsha Science and Technology Project (China) (No. kq2004137), and Chinese Anti-Cancer Association HER2 target Chinese Research Fund (No. CORP-239-S5).
