# Supplementary material for: CircAkap7: A novel circular RNA with a role in Doxorubicin-induced cardiotoxicity
Source: Genes Dis. 2024 Mar 2;12(1):101253. doi: 10.1016/j.gendis.2024.101253 (PMC12053662; doi:10.1016/j.gendis.2024.101253)

**Supplemental Tables:**

**Table S1** Primers of circRNAs and GAPDH

| RNA | F | R |
| --- | --- | --- |
| circCnot2 | AAGTTTATACGGGCAACAAAAGG | GAGTCGTGTCCTTTCCTCACAGTAC |
| circAkap7 | GAGTCTTCCATCGTGATCGGAA | GGTTCCAGTTCTCGTGCCACT |
| circLrrc58 | CAAACCCAAAGTGTGGTGTTTAG | AAGGCAGATTTGCTAATTCTGGTG |
| circZfp407 | AACTGCCCAAAATGCGACTATG | CATAAGTGGCATGTAAATTTCCAGC |
| circStk39 | AAGGCCCACAGCAGCAGAAC | AGTTGCTCCGCTGCCTTCT |
| circTtn | GTGGAGCACTATGAAGAGCATGA | GGAGGCACTTCATGTTCTTTTG |
| circCd36 | GCAAAGTTGCCATAATTGAGTCC | CACATTTCAGAAGGCAGCTGTG |
| circInsr | ATCGTTGGCCCTGTGACTCA | GCTTTCGGGATGGCCTCAT |
| circArhgap10 | CTCTGATGAGACCACAGGAAGAG | ACAGTCCTTGGTCATTTATACCTTT |
| circZbtb16 | CACAGACAGACCCATACTGTCCC | GGCTCTCTTTCCTTCTCTGCTCT |
| circSetd2 | GTCTCCTCATGATTGAATGGCAAA | CCGTCCCTGTTCCTCCAAAT |
| circPrdm5 | GCACATTCGTACTCACACCAG | AGGCTTGACACTGAGGTACA |
| circKat6b  (14:21516626\|21517498) | AGCGTGCTGTGAACAATGG | ACATCTTTAGGAAACCAAGACCTG |
| circSorbs1 | CAGAGCCTCCTGGATAAGTCG | TCTCCCAAACTCCAATTCCGA |
| circRab11a | ACGTCATCTCAGGGCAGTTC | CCAGAATCTCCAATAAGGACAACTG |
| circFbxw8 | CCGACGACTCAGCCAGAA | TCCCAAATGTTAAGGATCCCTTGT |
| circVdac2 | CTTCCAGCTACACACAAATGTGAAT | GTGTTATCGGTGTTCCACTTCT |
| circCnot6l | TGCCAGCATTGAAGGATCGT | GGAAGCTGCTCTGGATGAATTT |
| circCnksr3 | GGGCTCCATTCACAGGGATC | CAGGCAGTCATCCAACCCTT |
| circEhbp1 | AGATCTCTCTACTTCTCCAAAGAAC | TGGTATTCACAGTGGCCAAGT |
| circFkbp15 | ACAGATGGCCTCACAGGGG | TGGAGTCTGTTGGCTGAGT |
| circClasp1 | GCTCTCGATGTCACCAGAAG | TGCCGCAGATAATGGGGAAT |
| circCluh | TGCTCCGAGTTGTGGAAGATC | ATCTCCAGGCTCACCCTCAT |
| circMacf1 | ATCGCCCTGGACTTCCTAAAG | ACTCGGTCCCGCTCATCT |
| circKat6b  (14:21618891\|21637640) | GGACGCTTTCTCATTGATTTCAGA | GCATTTTTGCCCTGGACTCG |
| circKlc1 | TGACGGAGGGGAGGAAGGAT | TCTGCCCAGGGTTTTCTCG |
| circZeb1 | GCACTTACGGATTCACAGTGTTAC | CTGTCACTGCCTCCTGGTAAT |
| circVwa8 | CTTGGCGAACACTTACTATTGGT | AAGACAGGCTTTGGTAATGGC |
| circArhgap21 | CACAGAAGAAGGTGCCGAAGA | GCTTGAAATGGCTGCGTTG |
| circCrim1 | TCAACGGAGAACCTCACTGC | GCAGGCTGGCTTTGTTTCTT |
| GAPDH | GAACGGGAAGCTCACTGG | GCCTGCTTCACCACCTTCT |

**Table S2** Primers of miRNAs

| miRNA | MiRNA sequence | Forward primers of QPCR |
| --- | --- | --- |
| mmu-miR-327 | ACUUGAGGGGCAUGAGGAU | GACTTGAGGGGCATGAGGAT |
| mmu-miR-218-1-3p | AAACAUGGUUCCGUCAAGCACC | AAACATGGTTCCGTCAAGCACC |
| mmu-miR-497a-5p | CAGCAGCACACUGUGGUUUGUA | CAGCAGCACACTGTGGTTTGTA |
| mmu-miR-322-5p | CAGCAGCAAUUCAUGUUUUGGA | CAGCAGCAATTCATGTTTTGGA |
| mmu-miR-148b-5p | GAAGUUCUGUUAUACACUCAGGCU | GAAGTTCTGTTATACACTCAGGCT |
| mmu-miR-106a-5p | CAAAGUGCUAACAGUGCAGGUAG | CAAAGTGCTAACAGTGCAGGTAG |
| mmu-miR-93-5p | CAAAGUGCUGUUCGUGCAGGUAG | CAAAGTGCTGTTCGTGCAGGTAG |
| mmu-miR-350-3p | UUCACAAAGCCCAUACACUUUC | CGTTCACAAAGCCCATACACTT |
| mmu-miR-17-5p | CAAAGUGCUUACAGUGCAGGUAG | CAAAGTGCTTACAGTGCAGGTAG |
| mmu-miR-188-5p | CAUCCCUUGCAUGGUGGAGGG | GATCCCTTGCATGGTGGAGG |
| mmu-miR-192-3p | CUGCCAAUUCCAUAGGUCACAG | CTGCCAATTCCATAGGTCACAG |
| mmu-miR-9-5p | UCUUUGGUUAUCUAGCUGUAUGA | GGTCTTTGGTTATCTAGCTGTATGA |
| mmu-miR-27b-3p | UUCACAGUGGCUAAGUUCUGC | TTCACAGTGGCTAAGTTCTGC |
| mmu-miR-6982-5p | CUGGAGGAUCGCAGGGGUGGCCUGG | ATAAGGATCGCAGGGGTGGC |
| mmu-miR-18a-5p | UAAGGUGCAUCUAGUGCAGAUAG | CTAAGGTGCATCTAGTGCAGATAG |
| mmu-miR-20a-5p | UAAAGUGCUUAUAGUGCAGGUAG | CCGAGAGCTTATAGTGCAGGTAG |
| mmu-miR-291b-3p | AAAGUGCAUCCAUUUUGUUUGU | CCAGTGCATCCATTTTGTTTGT |
| mmu-miR-129-5p | CUUUUUGCGGUCUGGGCUUGC | CTTTTTGCGGTCTGGGCTTGC |
| mmu-miR-15b-5p | UAGCAGCACAUCAUGGUUUACA | TAGCAGCACATCATGGTTTACA |
| mmu-miR-207 | GCUUCUCCUGGCUCUCCUCCCUC | TTCTCCTGGCTCTCCTCCCT |
| mmu-miR-106b-5p | UAAAGUGCUGACAGUGCAGAU | CTAAAGTGCTGACAGTGCAGAT |
| mmu-miR-742-3p | GAAAGCCACCAUGCUGGGUAAA | GAAAGCCACCATGCTGGGTAAA |
| mmu-miR-687 | CUAUCCUGGAAUGCAGCAAUGA | CTATCCTGGAATGCAGCAATGA |

**Table S3** Divergent primers and convergent primers of circAkap7 and GAPDH

| RNA | F | R |
| --- | --- | --- |
| 18S | GGACAGGATTGACAGATTGATAGC | TGCCAGAGTCTCGTTCGTTA |
| U6 | GGAACGATACAGAGAAGATTAGC | TGGAACGCTTCACGAATTTGCG |
| circAkap7  (Convergent primer) | AAGGGAGTGAGAAAAATCGAGC | GATCACGATGGAAGACTCACA |
| circAkap7  (Divergent primer) | GAGTCTTCCATCGTGATCGGAA | GGTTCCAGTTCTCGTGCCACT |
| GAPDH  (Convergent primer) | GAACGGGAAGCTCACTGG | GCCTGCTTCACCACCTTCT |
| GAPDH  (Divergent primer) | GGTTGTCTCCTGCGACTTCA | CGGGGTAAGGGCAGCATTTA |
| Akap7 mRNA | TCGTGATCGGTGAGAAGGAC | TCACTGCCATCACCATTCCG |

**Table S4** Infection sequence of circAkap7

| Interference RNA | sequence |
| --- | --- |
| sh-circAkap7-1 | CATCGTGATCGGAAAACTA |
| sh-circAkap7-2 | CGTGATCGGAAAACTAGAT |
| sh-circAkap7-3 | GTGATCGGAAAACTAGATT |
| sh-NC | TTCTCCGAACGTGTCACGT |

**Supplemental Figures:**

**Figure S1 Identification of the DIC mice model and its differentially expressed circRNAs.** (A) Representative images of HE staining in myocardium, magnification, ×200. (B) Levels of CK, CK-MB, LDH, cTnT in serum. ^*^*P*<0.05, ^**^*P*<0.01 vs. Con. (C)Representative pictures of TUNEL staining in myocardium, magnification, ×200, apoptotic cardiomyocytes nuclei appear green fluorescence and normal nuclei appear blue fluorescence, the white array indicated the apoptotic cardiomyocytes. (D) Volcano plot of differentially expressed circRNAs, in which red, blue and gray dots represent significantly up-regulated, significantly down-regulated and no differentially expressed circRNAs, respectively.

**
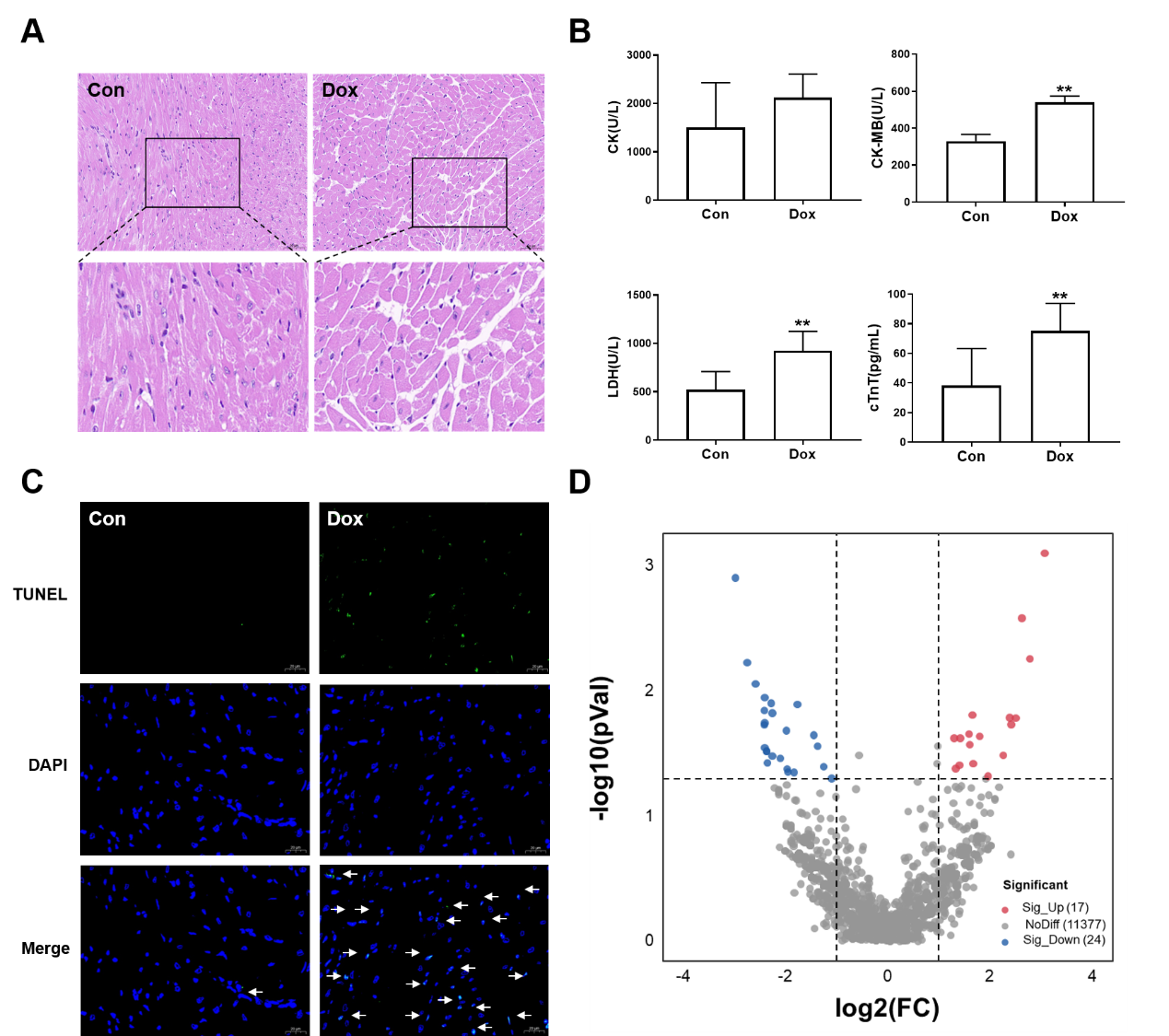
**

**Figure S2 Validation of differential expression of circRNAs in the DIC model.** (A) RT-QPCR in DIC cell model (n=3) verified the accuracy of up-regulated circRNA sequencing results. (B) RT-QPCR in DIC mouse model (n=10) verified the accuracy of up-regulated circRNA sequencing results. (C) Differential expression of down-regulated circRNAs in DIC cell model (n=3), and RT-QPCR verified the accuracy of down-regulated circRNA sequencing results. (D) Differential expression of down-regulated circRNAs in DIC mouse model (n=10), and RT-QPCR verified the accuracy of down-regulated circRNA sequencing results. ^*^*P*<0.05, ^**^*P*<0.01.


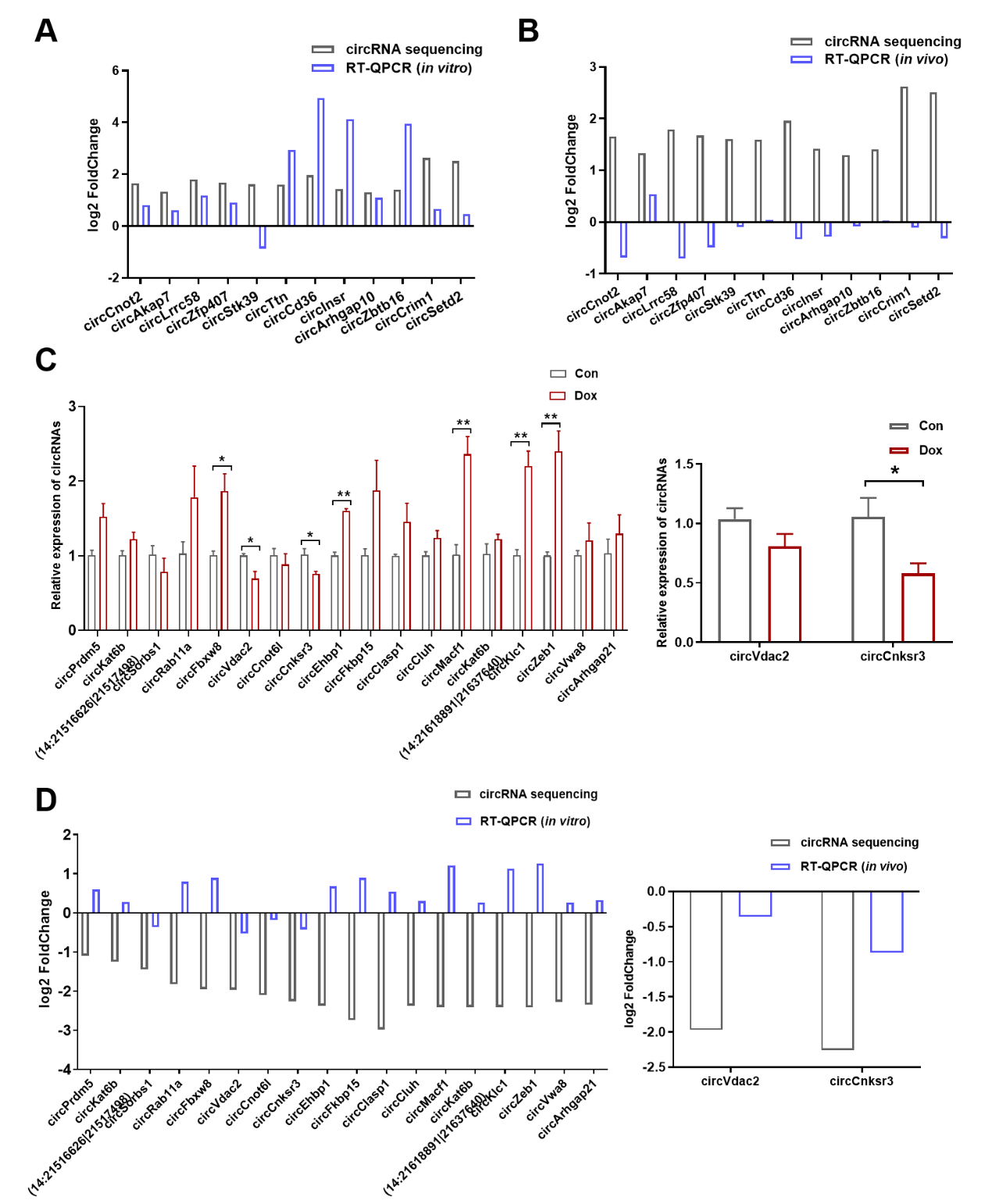


**Figure S3 Ring verification and nuclear location of circAkap7.** (A) The sequence of circAkap7. (B) circAkap7 was amplified based on cDNA and gDNA (
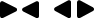
: convergent primers,
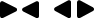
 : divergent primers). (C) Cellular RNA fractionation assay (n=3). (D). RNase R treatment assay. **P*<0.05, ***P*<0.01, n=3.


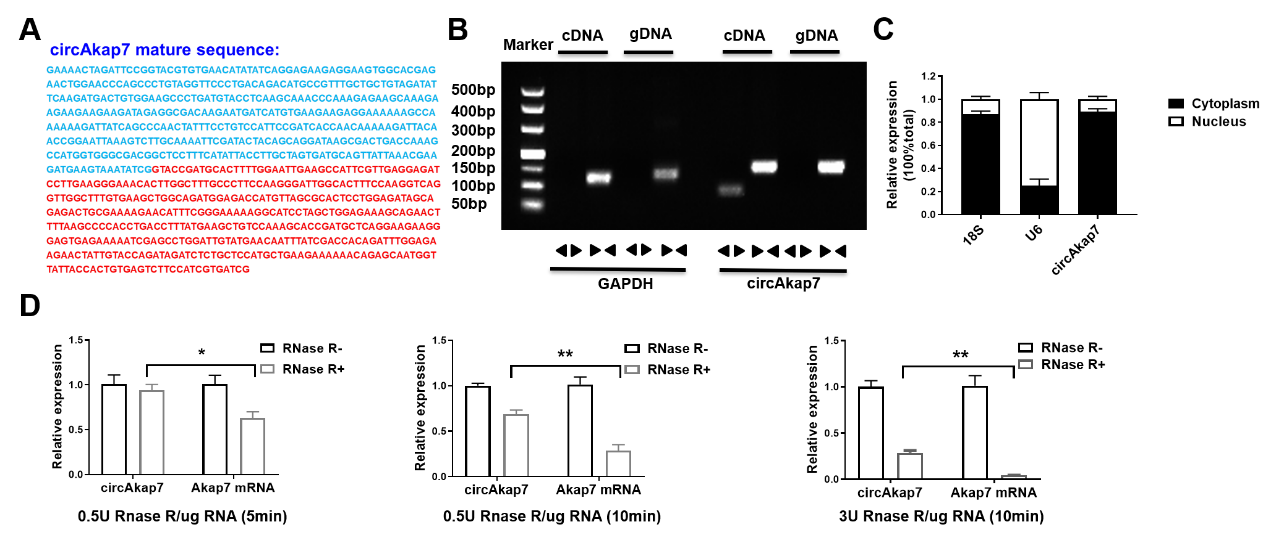


**Figure S4 Function experiments of circRNAs in DIC cell model.** (A) Lentiviral vector atlas of circAkap7 interference. (B) The relative expression of circAkap7 in Dox-treated circAkap7 knock-down cells. (C) The relative expression of Akap7mRNA in Dox-treated circAkap7 knock-down cells. (D) CCK8 assay detected cell viability in cells with circAkap7 knockdown.


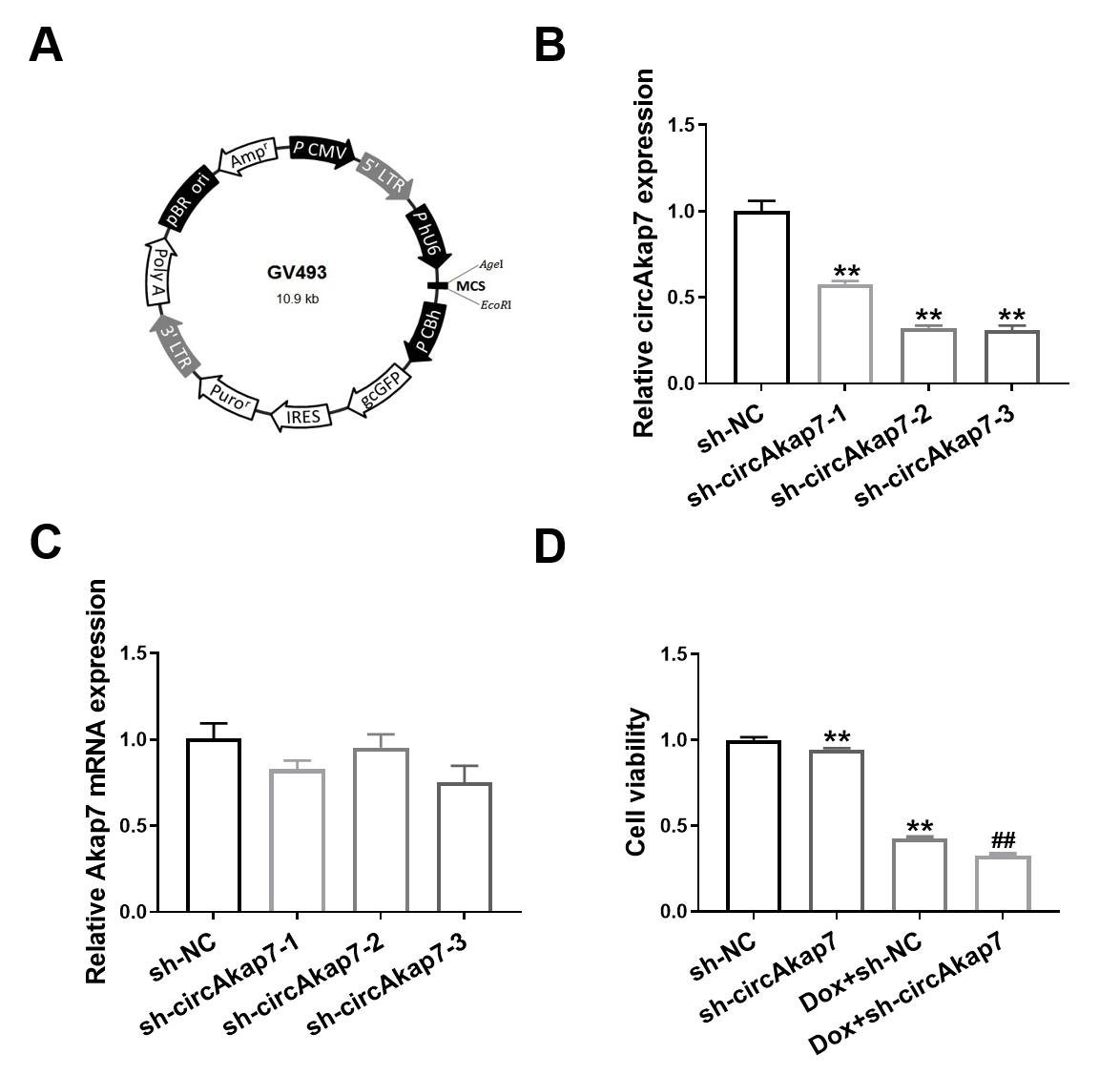


**Figure S5 The mRNA sequencing analysis of Dox-treated NC cells and circAkap7 knockdown cells.** (A) Correlation heat map. The redder the color, the higher the correlation between samples. (B) Volcano plot of differentially expressed mRNAs, in which red, blue and gray dots represent significantly up-regulated, significantly down-regulated and no differentially expressed mRNAs, respectively. (C) Principal component analysis. (D) GO enrichment analysis of differentially expressed genes. (E) KEGG enrichment analysis of differentially expressed genes. (F) GSEA analysis of differentially expressed genes, based on GO(a), KEGG(b) and Reactome(c) gene set respectively.


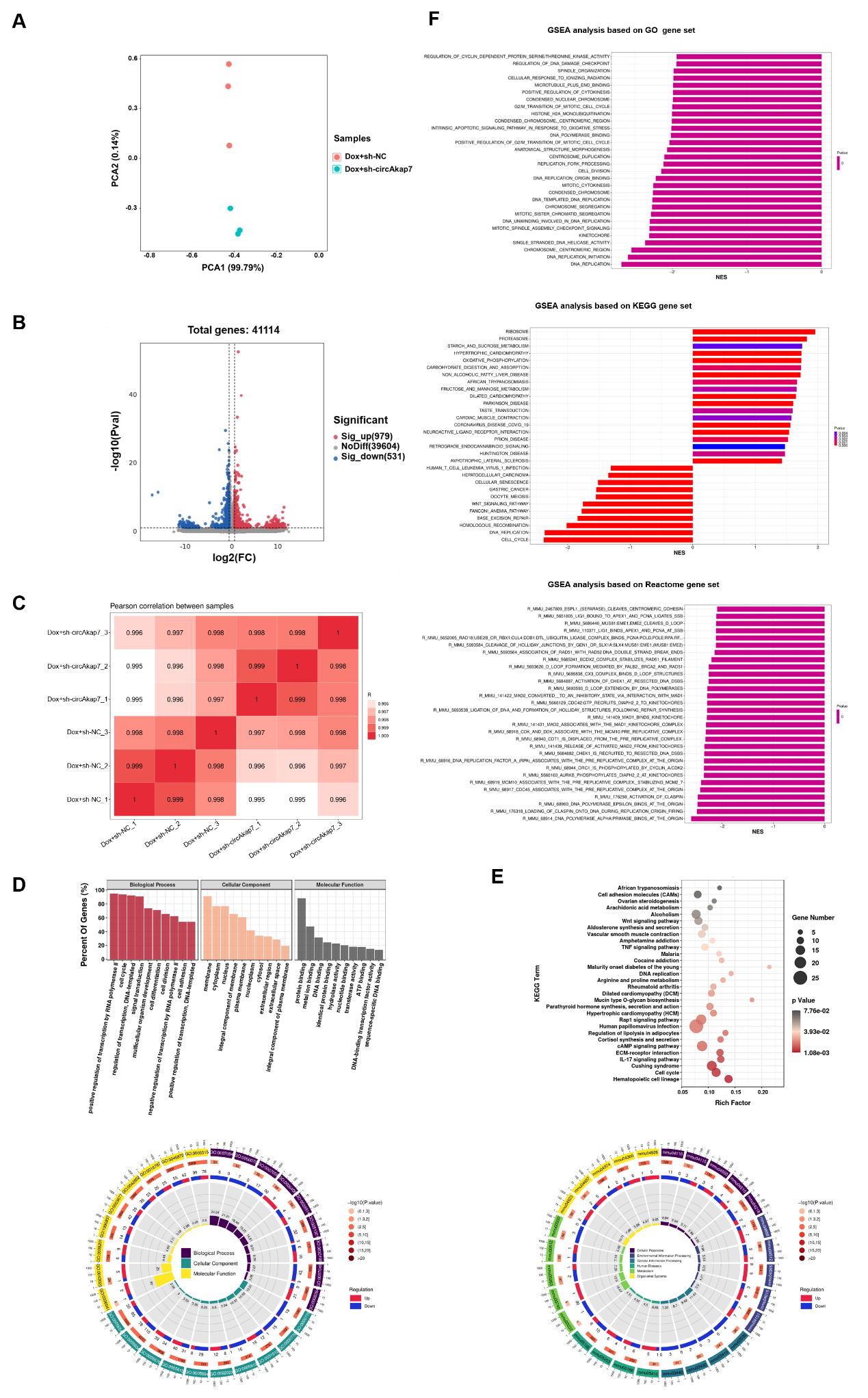


**Figure S6 PPI networks of differentially expressed genes.** (A) The original PPI network exported from STRING database (interaction score> 0.7) with 450 nodes and 1836 edges. (B) PPI network by importing original PPI network to Cytoscape 3.7.2 with 450 nodes and 1836 edges. (C) By CytoNCA, the core PPI network based on (Fig.1M) with 42 nodes and 634 edges. (D) By CytoHubba, a core PPI network from (B) containing the top 50 nodes with the largest MCC values containing 50 nodes and 72 edges. (E-G) By using MCODE, top three clusters extracted from (B) with the highest score of 33.756 (E), 5.667 (F), and 5.600 (G), respectively. (H) Venn diagram of core differentially expressed genes.


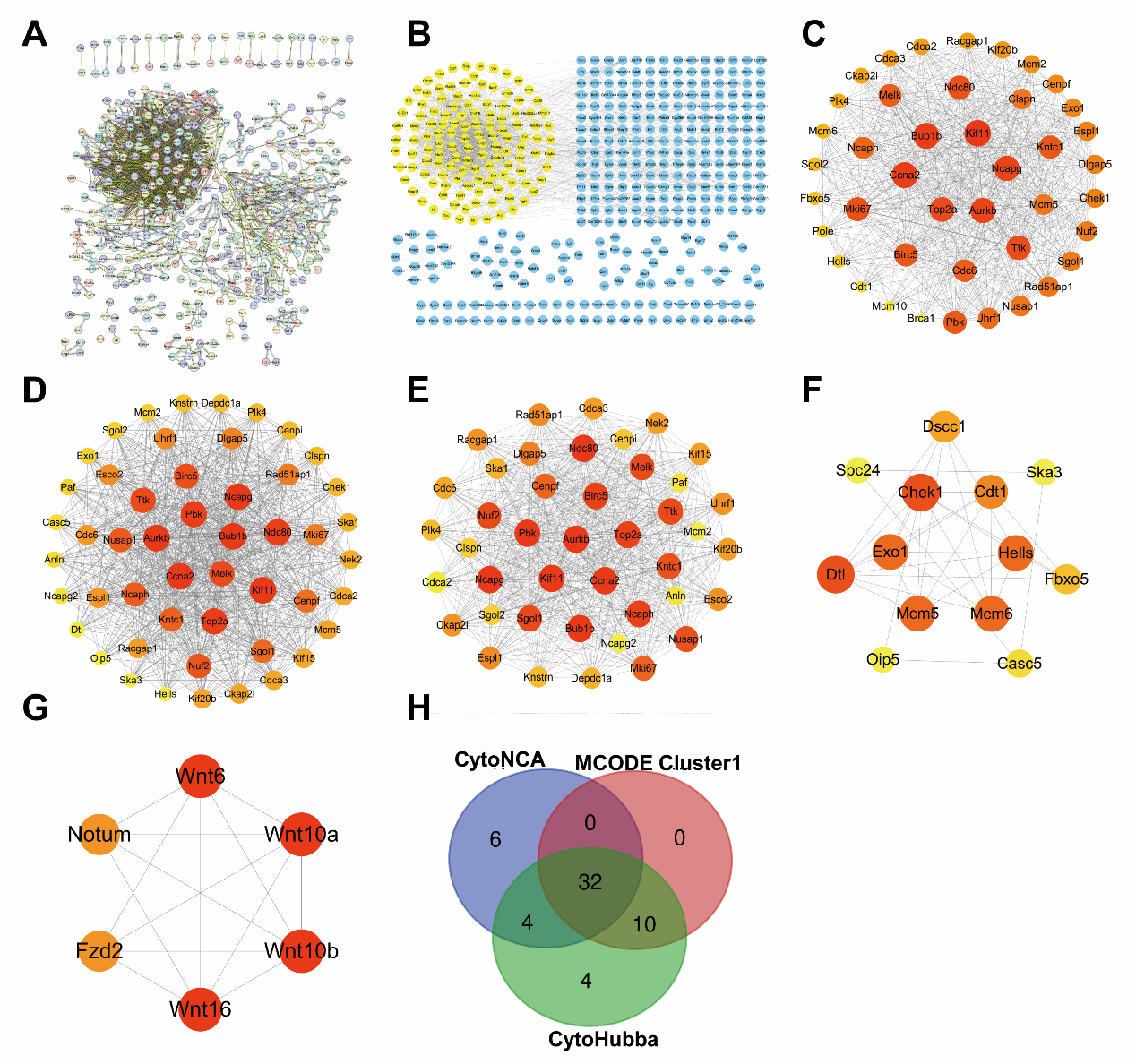


**Figure S7 Node degree distribution as well as degree values of genes in PPI networks.** (A) Node degree distribution of the PPI network in Figure S6B. (B) Node degree distribution of the PPI network in Figure 1M. (C) Node degree distribution of the core PPI network in Figure S6C. (D) Node degree distribution of the core PPI network in Figure S6D. (E) Node degree distribution of the core PPI network in Figure S6E. (F) Node degree distribution of the PPI network in Figure S6F. (G) Node degree distribution of PPI networks in Figure S6G. (H) Node degree values of the core PPI network from Figure S6C. (I) Node degree values of the core PPI network from Figure S6D. (J) Node degree values of the core PPI network from Figure S6E.


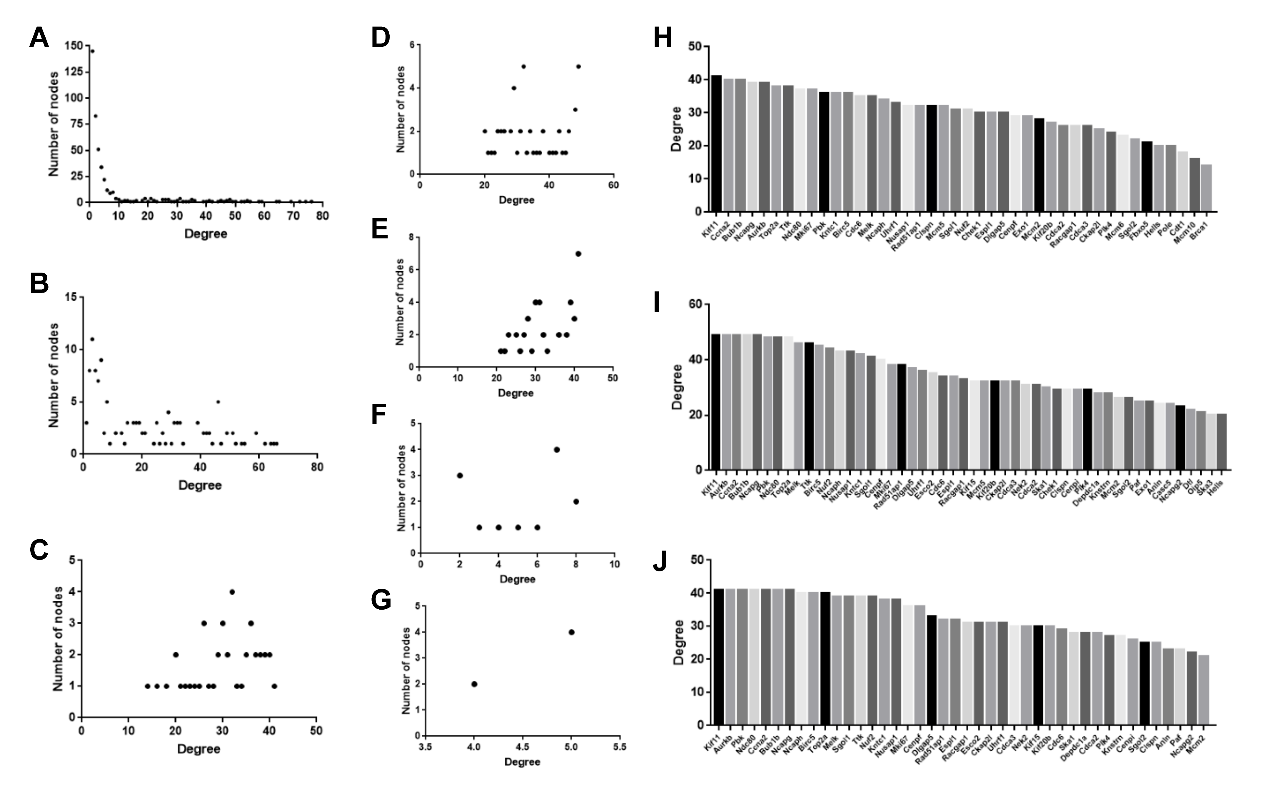


**Figure S8 circAkap7-miRNA-mRNA regulatory network construction.** (A) Prediction of target microRNAs for circAkap7. (B) Prediction of binding sites of 4 potential target miRNAs with circAkap7 by miRanda. (C-F) Screening of potential target mRNAs for mmu-miR-93-5p (C), mmu-miR-350-3p (D), mmu-miR-17-5p (E) and mmu-miR-291b-3p (F). (G-J) as well as potential target mRNAs for circAkap7-mmu-miR-93-5p axis (G), circAkap7-mmu-miR-350-3p axis (H), circAkap7- mmu-miR-17-5p axis (I) and circAkap7-mmu-miR-291b-3p axis (J).


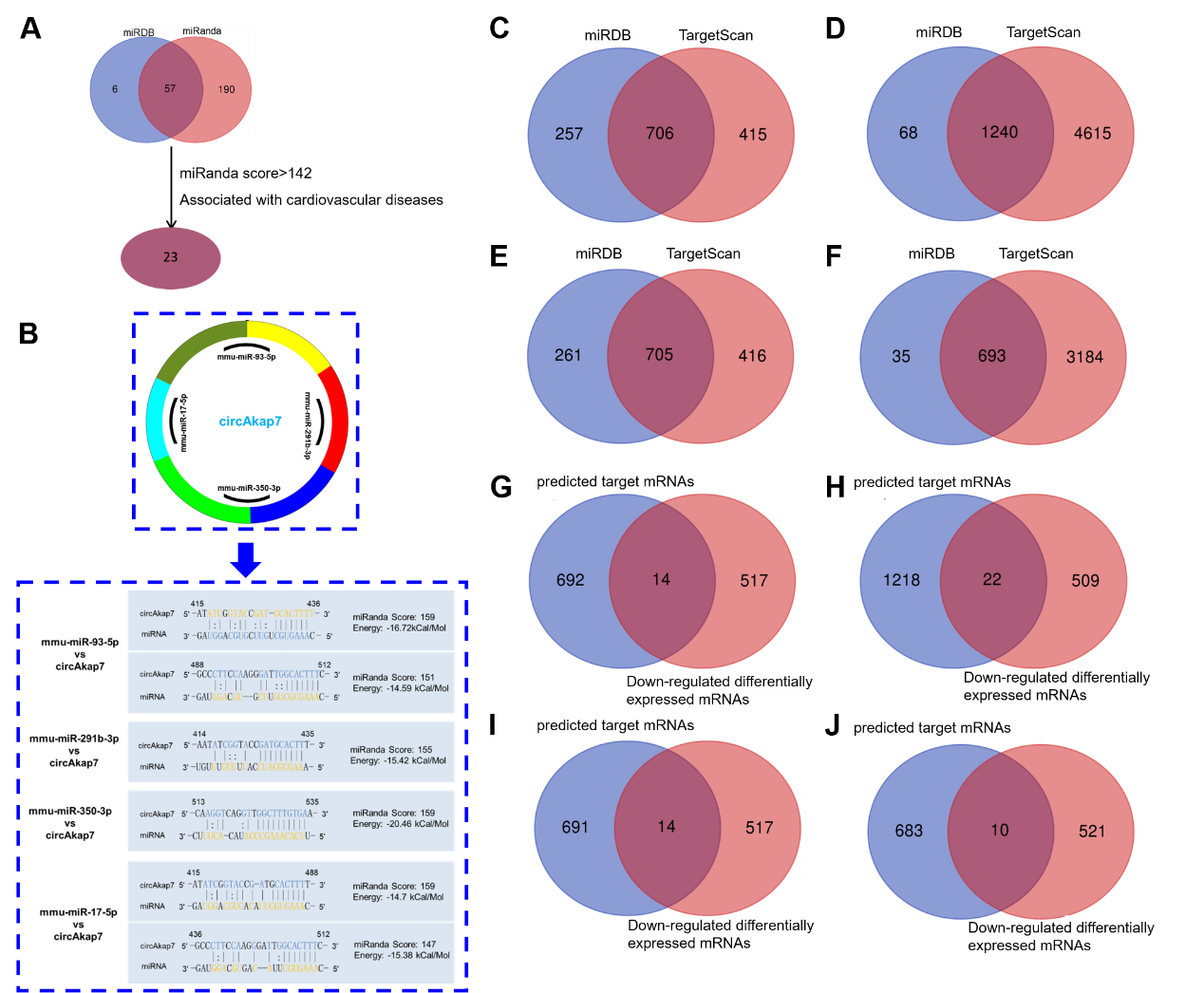

Supplement: Multimedia component 1 [file mmc1.docx]
